# Phytochemical Characterization and Evaluation of the Anticholinesterase and Anti-*Trypanosoma cruzi* Potential of Andean Amaryllidaceae from Bolivia: (*Pyrolirion boliviense* and *Stenomesson miniatum*)

**DOI:** 10.3390/life16071139

**Published:** 2026-07-09

**Authors:** María Lenny Rodríguez-Escobar, Vineet Singh Raj, Nieves Martínez-Peinado, Alfredo F. Fuentes, Carla Maldonado, Juan Carlos Gabaldón-Figueira, Julio Alonso-Padilla, Jaume Bastida, Luciana R. Tallini, Laura Torras-Claveria

**Affiliations:** 1 Secció de Fisiologia Vegetal, Departament de Biologia, Sanitat i Medi Ambient, Facultat de Farmàcia i Ciències de l’Alimentació, Universitat de Barcelona, Avda. Joan XXIII n° 27-31, 08028 Barcelona, Spain; m-rodriguez@ub.edu (M.L.R.-E.);; 2 ISGlobal, C/ Aiguader 88, 08003 Barcelona, Spain; singhvineet.2908@gmail.com (V.S.R.); nieves.martinez@ub.edu (N.M.-P.); juancarlos.gabaldon@isglobal.org (J.C.G.-F.); julio.a.padilla@isglobal.org (J.A.-P.); 3 CIBER de Enfermedades Infecciosas, Instituto de Salud Carlos III (CIBERINFEC, ISCIII), 28029 Madrid, Spain; 4 Secció de Parasitologia, Departament de Biologia, Sanitat i Medi Ambient, Facultat de Farmàcia i Ciències de l’Alimentació, Universitat de Barcelona, Avda. Joan XXIII n° 27-31, 08028 Barcelona, Spain; 5 Herbario Nacional de Bolivia (LPB), Instituto de Ecología, Facultad de Ciencias Puras y Naturales, Universidad Mayor de San Andrés, Calle 27 Cota Cota, La Paz 10077, Bolivia; alfrefuentes@gmail.com (A.F.F.); cmaldonado@fcpn.edu.bo (C.M.); 6 Department of Latin America, Missouri Botanical Garden, 4344 Shaw Blvd., St. Louis, MO 63110, USA; 7 Departament de Microbiologia, Hospital de la Santa Creu i Sant Pau, 08025 Barcelona, Spain; 8 INTERTRYP, Campus international de Baillarguet, Université de Montpellier, 34398 Montpellier, France

**Keywords:** alkaloids of Amaryllidaceae, *Stenomesson miniatum*, *Pyrolirion boliviense*, GC-MS, cholinesterase inhibition, *Trypanosoma cruzi*, bioactive natural products, Alzheimer’s disease, Chagas disease, cytotoxicity

## Abstract

The Amaryllidaceae family is a rich source of structurally diverse alkaloids with recognized neuroactive and antiparasitic properties. This study provides the first phytochemical and biological characterization of *Pyrolirion boliviense* and wild *Stenomesson miniatum* from Bolivia. Alkaloid extracts from bulbs and leaves were analysed by GC–MS and evaluated for acetylcholinesterase (AChE), butyrylcholinesterase (BuChE), and *Trypanosoma cruzi* inhibitory activities. Thirty-two Amaryllidaceae alkaloids were identified, with *P. boliviense* exhibiting greater alkaloid diversity (25 compounds) and *S. miniatum* a higher total alkaloid content (227.86 vs. 138.92 μg Gal/100 mg DW). *P. boliviense* bulb extracts showed the strongest cholinesterase inhibition (AChE IC_50_ = 6.07 ± 0.47 μg·mL^−1^; BuChE IC_50_ = 30.93 ± 1.17 μg·mL^−1^), whereas *S. miniatum* extracts displayed weaker AChE inhibition and no detectable BuChE activity. In anti-*T. cruzi* assays, bulb extracts were the most active, with *S. miniatum* showing an IC_50_ of 0.90 ± 0.15 μg·mL^−1^ (SI = 20.12) and selective anti-amastigote activity (IC_50_ = 1.42 ± 0.66 μg·mL^−1^; SI = 12.77). These findings identify Bolivian Andean Amaryllidaceae as promising sources of bioactive alkaloids with potential applications for Alzheimer’s disease and Chagas disease drug discovery.

## 1. Introduction

Plant-derived bioactive compounds have historically played a central role in the discovery of novel pharmacologically active agents. More than 50% of the drugs approved in recent decades are derived directly or indirectly from natural products or inspired by natural-product scaffolds [1]. Among these, alkaloids stand out for their chemical diversity and their wide spectrum of biological activities: from analgesic and antiparasitic effects to modulators of the central nervous system, thereby providing a remarkable therapeutic potential [2,3,4,5].

The characteristic alkaloids of the subfamily Amaryllidoideae, known as Amaryllidaceae alkaloids (AAs), are particularly relevant due to their restricted distribution and the complexity and diversity of their chemical structures and variety of biological activities. More than 600 compounds have been identified [2].

Alzheimer’s disease (AD) is a multifactorial neurodegenerative disorder mainly characterized by changes in memory, judgement, behaviour, emotions, and abstract thinking that ultimately interferes with physical control over the body. It is currently affecting 37 million people, and the number is expected to rise to 51 million in 2030 and 90 million in 2050. It is the leading cause of dementia (60–70% of the cases), which constitutes the seventh most common death cause worldwide [6,7].

Galanthamine, a well-known Amaryllidaceae alkaloid, exemplifies the therapeutic potential of phytochemical research on this family and highlights its value as a source of therapeutically relevant natural products. Galanthamine is approved for the palliative treatment of AD since 2002, and it is clinically used in mild to moderate stages of the disorder, owing to its ability to inhibit acetylcholinesterase (AChE), and thereby maintain acetylcholine levels in the brain, which are markedly reduced in patients with AD [8,9,10].

Galanthamine has been isolated from several natural sources, including *Galanthus nivalis* L. and *Leucojum aestivum* L. in Bulgaria, *Galanthus woronowii* Losinsk. in Russia, *Ungernia victoris* Vved. in Uzbekistan, *Lycoris radiata* (L’Hérit.) Herb. in China, and daffodils in Central and Western Europe. However, limitations such as unsuccessful cultivation and slow plant regeneration hinder the ability of these sources to meet the growing pharmaceutical demand [11,12]. To reduce dependence on natural sources, galanthamine can also be produced through chemical synthesis, and more potent derivatives have been developed. Nevertheless, the overall yield of the total synthesis of this alkaloid remains too low to be economically viable [11]. Thus, there is a need for new sources of galanthamine, especially in the American continent [13].

Chagas disease (CD), also known as American trypanosomiasis, is an anthropozoonosis caused by the protozoan parasite *Trypanosoma cruzi* (*T. cruzi*). Classified as a neglected tropical disease, CD is a complex health condition shaped by social and environmental determinants. Delayed diagnosis and insufficient treatment may lead to severe, life-threatening complications. More than seven million people are estimated to be infected worldwide, with more than 100 million individuals at risk, primarily in endemic regions of the Americas [14]. However, increased migration and urbanization have expanded its global distribution, with cases reported in 44 countries [14]. The World Health Organization recognizes CD as a global public health concern [14,15]. Transmission occurs mainly through contact with feces of *T. cruzi*-infected triatomine vectors. Infection can also occur through organ transplantation, blood transfusion, or vertical transmission from mother to child, the latter being of increasing relevance, especially in countries where the triatomine vector is absent. The disease progresses through an acute phase, often characterized as asymptomatic or presenting mild nonspecific symptoms, followed by an indeterminate chronic phase that can last for decades. However, 30–40% of infected individuals will progress to a symptomatic chronic stage characterized by cardiac and/or digestive tissue alterations. Moreover, epidemiological evidence suggests that chronic Chagas disease may be associated with cognitive impairment [16]. Only two anti-*T. cruzi* drugs are currently approved: benznidazole (BNZ) and nifurtimox (NFX) [14]. Both have several limitations, including reduced efficacy during the chronic phase of infection and prolonged treatment regimens frequently associated with adverse events that often lead to treatment discontinuation. Consequently, there is an urgent need for safer and more effective therapeutic options for the management of chronic CD. In this context, natural products represent a promising source for the identification and development of novel lead compounds [17].

South America represents an important center of diversity for Amaryllidaceae, particularly in the Andean region, where numerous endemic and geographically restricted species occur. Phylogenomic studies have clarified evolutionary relationships within Amaryllidoideae and identified distinct Andean lineages with well-defined morphological and biogeographic patterns [18,19]. However, Bolivian Amaryllidaceae remain poorly studied from a phytochemical perspective [18]. Bolivia is recognized as an important center of diversity for the genus *Hippeastrum*, with 35 recorded species, of which 24 are endemic [20]. Rodriguez-Escobar and coworkers provided the first report on the phytochemical characterization and biological evaluation of Bolivian Amaryllidoideae species [21].

Within this framework, *Pyrolirion* and *Stenomesson* belong to distinct tribal lineages and exhibit clearly differentiated morphological characteristics. According to Plants of the World Online [22], *Pyrolirion boliviense* (Baker) Sealy is endemic and restricted to Bolivia. It has been reported from Andean environments in the Department of La Paz, typically occurring on rocky slopes between approximately 2800 and 3200 m above sea level. The species exhibits erect, actinomorphic flowers with a tubular perianth and a deeply trifid style, consistent with its taxonomic placement. In contrast, *Stenomesson miniatum* (Herb.) Ravenna is distributed in Peru and western Bolivia and occurs primarily in Andean habitats above 2000 m, reaching elevations near 3500 m [22]. The species is characterized by pendulous, urceolate flowers and exserted stamens, distinguishing it from closely related genera. In Peru, it has been categorized as Vulnerable (VU) under national legislation, reflecting conservation concerns within part of its distribution range.

Despite their botanical relevance, phytochemical investigations of *P. boliviense* and *S. miniatum* remain limited compared with those of extensively studied genera such as *Hippeastrum*. To the best of our knowledge, only two studies describing the phytochemical characterization and evaluation of biological activity within the genus *Stenomesson* have been reported, both focusing on Ecuadorian species, specifically *Stenomesson aurantiacum* [23,24]. Only one study has reported the phytochemical characterization and evaluation of the cytotoxic and antibacterial activities of *S. miniatum* bulbs. However, the bulbs analysed in that study were obtained from a nursery, where environmental conditions differ from those experienced by wild populations [25]. Regarding the genus *Pyrolirion*, only a single study has been found addressing the phytochemical composition of the species *Pyrolirion albicans* [26] and no phytochemical or biological studies have been reported for *P. boliviense*.

Therefore, the present study aims to provide a phytochemical characterization and biological evaluation of *P. boliviense* and *S. miniatum*, with the evaluation of the inhibition of AChE and BuChE, and the antiparasitic activity against *T. cruzi*, contributing to the chemotaxonomic understanding of Amaryllidaceae and to the identification of alkaloid-rich extracts with potential neuroactive and/or antiparasitic properties.

## 2. Materials and Methods

### 2.1. Plant Material

**Figure 1:**
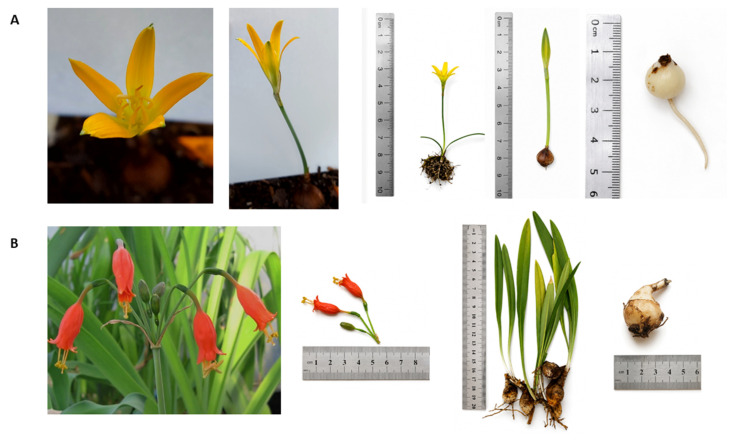
Morphological characteristics of *P. boliviense* (**A**) and *S. miniatum* (**B**) collected in Bolivia. Photographs were taken by M.L. Rodríguez-Escobar.

**Figure 2:**
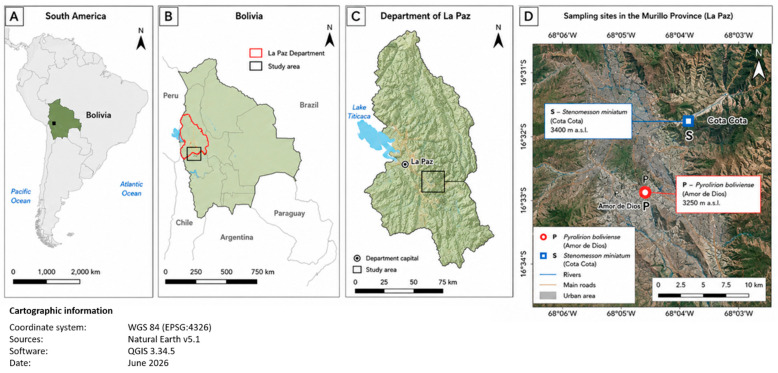
Geographic location of the sampling sites of *P. boliviense* (**P**) and *S. miniatum* (**S**). (**A**) shows Bolivia within South America. (**B**) shows the Department of La Paz. (**C**,**D**) show the sampling sites at Amor de Dios and Cota Cota.

The plant material used in this study corresponded to two Andean species of the Amaryllidaceae family, *S. miniatum* (Herb.) Ravenna and *P. boliviense* (Baker) Sealy (Figure 1), collected during the flowering period (February–March 2023) in the region of La Paz, Bolivia, as detailed in Figure 2. Specimens were taxonomically identified and validated by Dr. Alfredo Fuentes (Herbario Nacional de Bolivia–LPB), following the naming standards of Tropicos^®^ and Plants of the World Online (POWO). The reference specimens (vouchers) were deposited at the Herbario Nacional de Bolivia (LPB) as detailed in Table 1.

### 2.2. Alkaloid Extraction

The purified alkaloid extracts were prepared following the protocol reported by [21].

The fresh samples were cut and dried at 40 °C until constant weight, and then pulverized in a stainless-steel blade mill to a homogeneous powder. From each sample, 1 g of dry plant material (bulbs and leaves) was used, which was macerated in HPLC grade methanol (Fisher Scientific, Alcobendas, Spain) (50 mL) at 25 °C for three days, renewing the solvent every 24 h (50 mL/day) to optimize extractive efficiency.

During maceration, samples were subjected to ultrasonic bath treatment (20 min, four times a day) to promote the release of alkaloids. After each treatment, the mixture was filtered and the solvent was removed by evaporation at reduced pressure.

Each extract was acidified with 2% sulfuric acid (Carlo Erba, Rodano, Italy) (*v*/*v*; 50 mL) to adjust the pH to 2 and then treated with diethyl ether (Fisher Scientific, Alcobendas, Spain) (3 × 50 mL) to remove neutral compounds. The aqueous phase was alkalized at pH 9–10 with ammonium hydroxide (25%, *v*/*v*) (Carlo Erba, Rodano, Italy) and reextracted with ethyl acetate (Fisher Scientific, Alcobendas, Spain) (3 × 50 mL) to obtain the alkaloids.

After evaporation of the solvent, the dry alkaloid extract was used for chemical characterization and biological assays. Extraction yields are reported in Table 2.

### 2.3. GC-MS Analysis

For chromatographic analysis, 2 mg of each alkaloid extract were dissolved in 1 mL of codeine-containing methanol (25 μg·mL^−1^) as an internal standard. One μL of the mixture was injected into an Agilent 6890N gas chromatograph coupled to an Agilent 5975 mass spectrometer, equipped with a Series 7683B automatic injector (Agilent Technologies, Santa Clara, CA, USA).

A TR-45232 Sapiens-X5MS column (30 m × 0.25 mm × 0.25 μm) was used in splitless mode, with electron impact ionization (70 eV). The temperature program consisted of starting at 100 °C for 12 min, increasing to 180 °C at 15 °C·min^−1^ (1 min of maintenance), increasing to 300 °C at 5 °C·min^−1^ and final maintenance for 10 min. The injector and detector temperatures were 250 °C and 280 °C, respectively; carrier gas flow (He): 1 mL·min^−1^.

### 2.4. Identification and Quantification of Alkaloids

The identification of the alkaloids in the alkaloid extracts was carried out by comparing the mass spectra and retention indices (RI) obtained with the reference values contained in the library of Amaryllidaceae alkaloids developed by the Natural Products Research Group of the Faculty of Pharmacy of the University of Barcelona, characterized by spectroscopic techniques (^1^H NMR, ^13^C NMR, CD, MS and IR) [27].

Chromatograms were processed and spectra deconvoluted using the AMDIS (Automated Mass spectral Deconvolution and Identification System, NIST) software, which allowed the identification of the peaks corresponding to the main and secondary alkaloids in each extract.

The relative quantification of alkaloids was determined using a galanthamine calibration curve (10, 20, 40, 60, 80, and 100 μg·mL^−1^) with codeine (25 μg·mL^−1^) as the internal standard. The area of the deconvoluted peaks was used for the relative quantification using Excel 2016 software. It should be noted that this quantification does not represent an absolute concentration values, but a comparative estimate of the relative content of alkaloids between different samples processed under identical experimental conditions, allowing the detection of variations in the chemical profile between plant species and organs to be analysed [21,28,29,30].

### 2.5. AChE and Butyrylcholinesterase (BuChE) Inhibition Assays

The evaluation of the inhibitory activity of alkaloid extracts on the enzymes AChE and butyrylcholinesterase (BuChE) was carried out following a colorimetric procedure based on the reaction of [31], adapted to microplate and optimized according to [21,32].

The assay is based on the formation of the yellow anion 5-thio-2-nitrobenzoate, generated by the reaction between 5,5′-dithiobis-(2-nitrobenzoic acid) (DTNB) and the hydrolysis product of acetylthiocholine or butyrylthiocholine, in the presence of the corresponding enzymes.

The enzymatic solutions were prepared from *Electrophorus electricus* AChE and equine serum BuChE (518 U·mL^−1^), stored at −20 °C until use. The reagents (DTNB, acetylthiocholine iodide (ATCI) and butyrylthiocholine iodide (BTCI) were acquired from Merck (Darmstadt, Germany).

In each assay, 50 μL of the enzyme solution (6.24 U·mL^−1^) were mixed with 50 μL of the alkaloid extract (PH, PB, SH or SB), previously dissolved in 8 mM phosphate buffer (pH 7.5, with 0.15 M NaCl). The mixture was incubated for 30 min at 25 °C. Subsequently, 100 μL of the substrate solution (DTNB 0.2 mM and ATCI or BTCI in phosphate buffer 0.1 M, pH 7.5) were added.

The formation of the colored complex was monitored at 405 nm using a Labsystem microplate reader (Helsinki, Finland). Residual enzyme activity was expressed as a percentage of the control without inhibitor.

Galanthamine was used as a reference inhibitor, evaluated at concentrations of 0.1–2.0 μg·mL^−1^ for AChE and 1–15 μg·mL^−1^ for BuChE, to determine the sensitivity of the assay.

Each sample was analyzed in independent triplicate. The dose-response curves were adjusted using the GraphPad Prism 9 software, obtaining the values of IC_50_ (mean inhibitory concentration). Results were expressed as mean ± standard deviation (SD). Statistical differences between treatments and positive control were assessed by one-way ANOVA, followed by Dunnett’s test (*p* < 0.05).

### 2.6. Host Cells and T. cruzi Cultures

Vero cells (green monkey kidney epithelial cells) and LLC-MK2 (rhesus monkey kidney epithelial cells) were maintained in DMEM (Fisher Scientific, Alcobendas, Spain) supplemented with 10% fetal bovine serum (FBS) (Biowest, Nuaillé, France) and 1% penicillin-streptomycin (Fisher Scientific, Alcobendas, Spain) as previously described [17]. *T. cruzi* parasites of the Tulahuen strain (DTU VI), genetically modified to express the β-galactosidase enzyme from *Escherichia coli*, were maintained in culture with LLC-MK2 cells as hosts in DMEM supplemented with 2% FBS and 1% penicillin-streptomycin-glutamine (Fisher Scientific, Alcobendas, Spain) as previously reported [17].

### 2.7. T. cruzi Growth Inhibition, Vero Cell Toxicity and Anti-Amastigote Assays

The evaluation of the antiparasitic and cytotoxic activity of the alkaloid extracts from *P. boliviense* and *S. miniatum* was performed as previously described by [17,33]. All the extracts were previously dissolved in dimethyl sulfoxide (DMSO) (Sigma-Aldrich, St. Louis, MO, USA) and later added to test plates at a starting concentration of 50, 125 or 250 μg·mL^−1^ and diluted following a ½ dose-response pattern. BNZ, the standard reference drug for CD treatment, was used as positive control of parasite growth inhibition, together with the corresponding positive and negative controls previously described [17].

For the anti-*T.* *cruzi* assay, Vero cells and trypomastigote solutions were each adjusted to a concentration of 1 × 10^6^ cells·mL^−1^ and combined at a 1:1 (*v*/*v*) ratio. Subsequently, 100 µL were added per well, yielding 5 × 10^4^ cells of each population (MOI = 1). After incubation for 96 h at 37 °C, chlorophenol red-β-D-galactopyranoside (CPRG) (Santa Cruz Biotechnology, Dallas, TX, USA) was added, and absorbance at 590 nm was measured to quantify β-galactosidase activity as a surrogate marker of parasite growth [17].

For the anti-amastigote assay, 5 × 10^6^ Vero cells were seeded into T-175 flasks and incubated for 24 h to allow monolayer formation. The cultures were subsequently washed with PBS, and purified trypomastigotes were added at 1 × 10^7^ parasites per flask (MOI = 1, considering a Vero cell duplication time of 24 h). After a 18 h incubation, the cell cultures were washed, detached and adjusted to a concentration of 5 × 10^6^ cells·mL^−1^. This solution was then used to seed 5 × 10^4^ cells per well in a 96-well plate already containing the extracts. These plates were then incubated at 37 °C for 72 h, after which CPRG was added for photometric readout [33].

For the Vero cell toxicity assay, the cells were diluted to 5 × 10^5^ cells·mL^−1^ and 100 µL were dispensed per well (5 × 10^4^ cells) in a 96-well plate containing the extracts. Following a 96 h incubation at 37 °C, cell viability was determined by quantifying fluorescence after the addition of a resazurin-based reagent (AlamarBlue) (Fisher Scientific, Alcobendas, Spain) [17].

Absorbance and fluorescence values were normalized to the positive growth controls. TC_50_ (half maximal toxicity concentration) and IC_50_ (half maximal inhibitory concentration for *T. cruzi* growth) values were determined as previously described [17]. The selectivity index (SI) was defined as the ratio between the TC_50_ and IC_50_, with a SI > 10 indicating selectivity towards the parasite over mammalian host cells.

All experiments were performed triplicate (*n* = 3), with variance expressed as mean ± standard deviation (SD).

## 3. Results and Discussion

### 3.1. Identification and Quantification of Bioactive Compounds

The alkaloids identified and quantified in *P. boliviense* and *S. miniatum* species are detailed in Table 3.

A total of 32 alkaloids were identified across the Bolivian species studied, with 25 detected in *P. boliviense* and 18 in *S. miniatum*, indicating a greater alkaloid diversity in *P. boliviense*. However, the total alkaloid content was higher in *S. miniatum* (227.86 µg Gal/100 mg DW) than in *P. boliviense* (138.92 µg Gal/100 mg DW), primarily due to the markedly elevated concentration of homolycorine in leaf samples (SH) (92.09 µg Gal/100 mg DW). The identified alkaloids were classified into different structural types according to [2]: lycorine-, homolycorine-, galanthindole-, crinine/haemanthamine-, narciclasine-, pretazettine-, montanine-, galanthamine-, and ismine-type alkaloids, as well as other alkaloid types.

The tissue-specific distribution of alkaloids differed between species. In *P. boliviense*, alkaloid accumulation was higher in bulbs (80.67 µg Gal/100 mg DW), whereas in *S. miniatum*, leaves exhibited the highest alkaloid content (159.50 µg Gal/100 mg DW).

*S. miniatum* is characterized by a markedly higher accumulation of homolycorine-type alkaloids compared to *P. boliviense*. The amount of homolycorine is high in leaves (SH) (92.09 µg Gal/100 mg DW), representing the most abundant structural group in the entire analysis. *S. miniatum* is also characterized by a notable amount of pretazettine-type alkaloids (42.57 µg Gal/100 mg DW), which are almost missing in *P. boliviense*. This group of alkaloids is dominated by a notable amount of tazettine in leaves (SH) (24.03 µg Gal/100 mg DW). *S. miniatum* also shows an important amount of crinine-haemanthamine-type alkaloids (38.55 µg Gal/100 mg DW) such as vittatine/crinine and haemanthamine with values ranging from 4.32 µg Gal/100 mg DW of haemanthamine to 5.69 µg Gal/100 mg DW of vittatine/crinine in SH. In this species, leaves (SH) also show the most diversity of crinine/haemanthamine-type alkaloids.

In contrast, *P. boliviense* shows comparatively lower values for these groups but exhibits a broader quantitative distribution across multiple structural types. The alkaloid profile of *P. boliviense* alkaloid is dominated by galanthamine-type alkaloids (34.63 µg Gal/100 mg DW), specially for the high amount of galanthamine (9.33 µg Gal/100 mg DW) in leaves (PH), and lycorine-type compounds (32.26 µg Gal/100 mg DW), with lycorine and 11,12-dehydroanhydrolycorine as the most abundant alkaloids (5.82 and 5.26 µg Gal/100 mg DW in PH, respectively). *P. boliviense* also contained appreciable amounts of alkaloids belonging to other structural types (24.03 µg Gal/100 mg DW), a group which is absent in *S. miniatum*, and basically represented by tyramine and demethylmesembrenol (5.64 and 5.31 µg Gal/100 mg DW in PH respectively). Finally, similarly to *S. miniatum*, *P. boliviense* also exhibited a diverse array of crinine/haemanthamine-type alkaloids with a total of 20.01 µg Gal/100 mg DW. This group was predominantly represented by 8-*O*-demethylmaritidine (5.27 µg Gal/100 mg DW in PH samples) and haemanthamine (5.24 µg Gal/100 mg DW in PH samples).

To date, only one other species of the genus *Pyrolirion* has been phytochemically investigated, *Pyrolirion albicans* from Peru [26]. Similar to *P. albicans*, *P. boliviense* shows a greater diversity of alkaloids in the bulb tissue. However, whereas *P. boliviense* is characterized by the presence of galanthamine-type alkaloids and a very low abundance of montanine-type compounds, *P. albicans* exhibits an alkaloid profile dominated by montanine-type alkaloids, particularly montanine and pancracine [26].

To our knowledge, only three phytochemical studies have been conducted on the genus *Stenomesson*. Two of them were focused on the same species, *Stenomesson aurantiacum* from Ecuador [23,24]. In the first study, samples were collected at Cuicocha Lake (Ecuador) [23], whereas in the second study samples were collected in Loja Province (Ecuador) [24]. The two studies reported different alkaloid profiles: [23] described a composition characterized by a particularly high abundance of haemanthamine, followed by tazettine, while Acosta [24] reported high levels of 11-hydroxyvittatine and galanthamine. The alkaloid profile of *S. miniatum* reported in the present study (Table 3) is characterized by a predominance of homolycorine, followed by tazettine. The relatively high abundance of tazettine is consistent with previous reports for *S. aurantiacum* [23]. Lianza [25] evaluated the alkaloid profile of bulb extracts from nursery-grown *S. miniatum*. In that study, several alkaloids were identified but not quantified. Among them, tazettine was detected, which is consistent with the results of the present study. However, homolycorine, found in high abundance in the present study, was not reported. These differences may be attributed to variations in analytical methodology, as well as to differences in the plant tissues analysed and in the origin of the plant material. For example, environmental conditions are known to play an important role in the synthesis and distribution of Amaryllidaceae alkaloids among plant tissues [34].

### 3.2. AChE and BuChE Inhibition Assays

The inhibitory activity of the alkaloid extracts from *P. boliviense* and *S. miniatum* is presented in Table 4.

Samples of *P. boliviense* showed markedly stronger inhibitory activity against AChE and BuChE than those of *S. miniatum*, with AChE IC_50_ values ranging from 6.07 to 24.86 μg·mL^−1^ and from 32.93 to 49.46 μg·mL^−1^, respectively.

In addition, BuChE inhibitory activity was detected only in *P. boliviense*, with an IC_50_ value of 30.93 μg·mL^−1^. From a pharmacological perspective, this BuChE-selective activity observed in *P. boliviense* is particularly relevant. Selective inhibition of BuChE has been associated with improved efficacy in increasing ACh levels, as BuChE is responsible for 40–90% of ACh metabolism in the later stages of AD, whereas under normal conditions it accounts for approximately 20% [4,35].

Although both species showed AChE inhibitory activity, clear quantitative differences were observed between plant organs. Bulb extracts (PB and SB) exhibited stronger inhibition (AChE IC_50_ values of 6.07 and 32.97 μg·mL^−1^, respectively) than leaf extracts (PH and SH; AChE IC_50_ values of 24.86 and 49.46 μg·mL^−1^, respectively).

Bulb extracts of *P. boliviense* (PB) exhibited the strongest cholinesterase inhibitory activity, with IC_50_ values of 6.07 μg/mL for AChE and 30.93 μg/mL for BuChE, respectively. Inhibitory activity was considerably lower in the leaf extract (PH) (IC_50_ of 24.86 μg/mL) and in *S. miniatum* extracts (SH and SB) (IC_50_ of 49.46 and 32.93 μg/mL, respectively). The strong AChE and BuChE inhibitory activity of PB extract correlates with alkaloid composition. This extract contains several galanthamine-type alkaloids including galanthamine, *N*-demethylgalanthamine, narwedine and 3-*O*-acetylgalanthamine, which are almost absent in *S. miniatum* extracts. Interestingly, the total content of galanthamine-type alkaloids was higher in PH (20.34 µg Gal/100 mg) than in PB (14.29 µg Gal/100 mg). Both *P. boliviense* extracts contained galanthamine. However, PH, which exhibited weaker cholinesterase inhibitory activity, contained approximately twice the galanthamine content of PB. This observation may be explained by the fact that several other galanthamine-type alkaloids have been reported to exhibit cholinesterase inhibitory activity, in some cases even greater than that of galanthamine [13,32,36]. Notably, PB contains 3-*O*-acetylgalanthamine, a galanthamine-type alkaloid absent in PH. Although no reports describing its individual cholinesterase inhibitory activity have been found in the literature, molecular docking studies have identified it as a promising ligand for AChE, exhibiting strong binding affinities toward five different AChE binding sites, in some cases even exceeding those reported for galanthamine [29]. Furthermore, this alkaloid was reported exclusively in *N. obsoletus* extracts in a study assessing the AChE and BuChE inhibitory activity of ten *Narcissus* species, where *N. obsoletus* showed the strongest inhibition of both enzymes [37].

Lycorine-type alkaloids have also been reported to exhibit AChE inhibitory activity [32,38]. These alkaloids are particularly abundant in PB and are almost absent in PH and in *S. miniatum* extracts. Therefore, the high content of this group of compounds in PB may contribute to its elevated inhibitory activity. In addition, 1-*O*-acetyllycorine, detected only in PB, has been reported to inhibit AChE more strongly than galanthamine [38], which may further contribute to the high inhibitory activity observed for PB.

### 3.3. T. cruzi Growth Inhibition Activity

**Figure 3:**
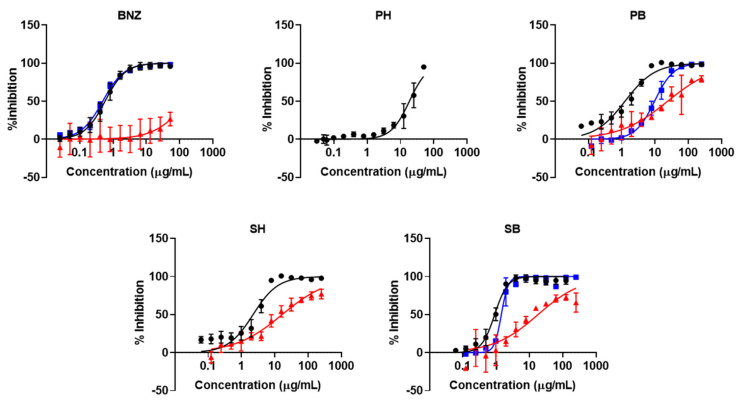
Dose-response curves of *P. boliviense* and *S. miniatum* extracts. Anti-*T. cruzi* activity is represented by black circles, Vero cell toxicity by red triangles and anti-amastigote activity by blue squares.

Results corresponding to the evaluation of the anti-*T. cruzi* activity of alkaloid extracts from *P. boliviense* and *S. miniatum* are summarised in Table 5. Dose-response curves of anti-*T. cruzi* activity, vero cell toxicity and anti-amastigote activity are shown in Figure 3.

*P. boliviense* leaves (PH) were considered inactive due to having an IC_50_ value > 5 × BNZ IC_50_. Although *S. miniatum* leaves (SH) showed a low IC_50_ (2.22 ± 1.03 μg·mL^−1^), the SI was <10 indicating significant host cell toxicity and the lack of a specific anti-*T. cruzi* response. Therefore, leaf extracts (PH and SH) were discarded from further progression. In comparison, bulb extracts of both species (PB and SB) exhibited the highest anti-*T. cruzi* activity. *S. miniatum* (SB) showed an IC_50_ of 0.90 ± 0.15 μg·mL^−1^ with an SI of 20.12, while *P. boliviense* (PB) had an IC_50_ of 1.25 ± 0.51 μg·mL^−1^ and a SI of 19.11. Then, when evaluated against the replicative amastigote forms, only the SB extract exhibited selective activity, displaying an IC_50_ of 1.42 μg·mL^−1^ and a SI of 12.77.

With respect to the alkaloid composition of SB, several of the identified alkaloids (homolycorine, crinine, 8-*O*-demethylmaritidine, crinamine, haemanthamine, tazettine, epimacronine, and galanthamine) have previously been reported as inactive against *T. cruzi* [17,39]. Notably, among the alkaloids detected across the extracts, only haemanthidine (identified exclusively in *Pyrolirium* extracts) was reported to exhibit activity against the parasite [39].

Among the alkaloids that have not yet been evaluated against *T. cruzi*, particular attention should be given to incartine, 11,12-dehydroanhydrolycorine, nerinine, galanthindole, trisphaeridine, and *O*-methyltazettine. In order to rationalize the selective activity of SB against amastigote forms, comparison with the alkaloid profile of PB provides useful insight. This comparison reveals a relatively high abundance of nerinine, as well as the exclusive presence of incartine and *O*-methyltazettine in the SB extract.

**Figure 4:**
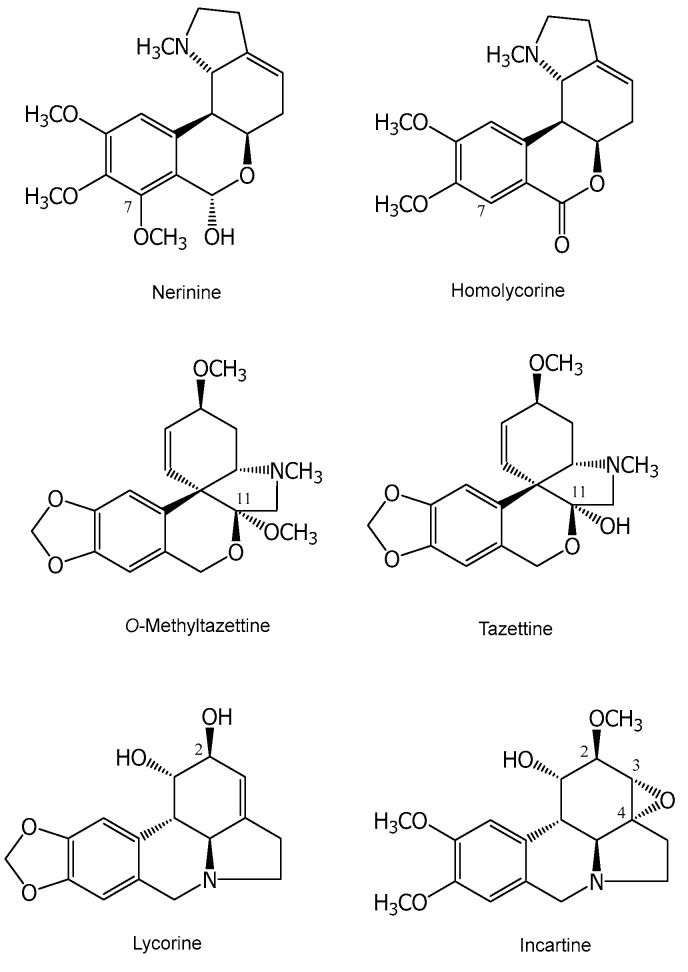
Structures of nerinine, homolycorine, *O*-methyltazettine, tazettine, lycorine and incartine.

Nerinine, a homolycorine-type alkaloid present in extracts PB and SB, is structurally related to the previously reported inactive compound homolycorine [39], differing mainly by the presence of a methoxy group at the C7 position (Figure 4). It is noteworthy that candimine, another homolycorine-type alkaloid that also contains a methoxy group at C7, displayed selective activity against the amastigote stage [40]. Therefore, it would be interesting to investigate whether this substituent difference could explain the activity observed.

Similarly, *O*-methyltazettine which is present in active extract SB differs from tazettine in the addition of a methoxy group at C11 (Figure 4). Tazettine has been also previously reported as inactive against *T. cruzi* [17]. The contribution of methoxy substituents to the activity of several compounds against *T. cruzi* and other trypanosomatids, including *T. brucei* and *Leishmania mexicana*, has been documented [41,42]. Hence, the presence of this structural group could be contributing to binding to a hydrophobic active site of the target protein, while also improving steric complementarity and optimizing orientation.

Incartine, detected in the active extract SB, differs structurally from lycorine by the presence of a methoxy group at C-2 and an epoxy ring at C3–C4 and by the presence of methoxy groups at C-8 and C-9 instead of a methylenedioxy group. (Figure 4). While lycorine has been reported to exhibit potent antiparasitic activity (IC_50_ = 0.70 µM), its pronounced cytotoxicity toward Vero cells results in a low SI (SI = 7.44) in our assay [17]. Therefore, it would be interesting to assess whether these structural differences could reduce cytotoxicity towards Vero cells while maintaining or enhancing anti-*T. cruzi* activity. In any case, a possible synergistic effect between alkaloids should also be considered.

## 4. Conclusions

The present study provides the first phytochemical and biological characterization of *P. boliviense* and wild populations of *S. miniatum* from Bolivia. A total of 32 Amaryllidaceae alkaloids were identified by GC–MS analysis, revealing marked interspecific and tissue-specific differences in alkaloid composition and distribution.

Both species exhibited inhibitory activity against AChE. *P. boliviense* samples were more potent than *S. miniatum* extracts and furthermore demonstrated selective inhibition of BuChE, which is of special pharmacological interest considering the increasing role of BuChE in advanced stages of Alzheimer’s disease.

Although leaf samples of both species resulted inactive in *T. cruzi* inhibition, bulb extracts showed anti-*T. cruzi* activity and particularly *S. miniatum* bulbs demonstrated selective activity against the replicative amastigote forms. Regarding alkaloid structures of active extracts, nerinine, incartine and *O*-methyltazettine could be responsible for the anti-amastigote activity of SB extract.

These findings highlight the chemical diversity and pharmacological potential of Bolivian Amaryllidoideae species, underscoring their potential for drug discovery and the development of functional foods. Furthermore, this research provides innovative parasite management strategies, extending its relevance beyond basic science. In addition, these results expand current knowledge of Andean genera such as *Hippeastrum*, *Clinanthus*, and *Rauhia*, contribute to the chemotaxonomic understanding of the genera *Stenomesson* and *Pyrolirion*, and highlight their importance as natural sources of lead molecules for neurodegenerative and neglected tropical diseases.

## Figures and Tables

**Table 1:** Botanical and geographical characterization of *P. boliviense* and *S. miniatum* specimens collected in the Department of La Paz, Bolivia. The geographical locations correspond to Andean valleys in the Murillo Province (La Paz Department), characterized by temperate semi-humid climate and well-drained soils typical of inter-Andean vegetation zones. AF, Alfredo Fuentes; LPB, Herbario Nacional de Bolivia.

Code	Species	Sample Type	Collector	Voucher	Geographical Location	Elevation (m.a.s.l.)	Annual Precipitation (mm)
PH	*P. boliviense* (Baker) Sealy	Leaves	AF	Alfredo F. Fuentes–23,942–LPB	La Paz (Amor de Dios), Bolivia	3250	400–600
PB	*P. boliviense* (Baker) Sealy	Bulbs	AF	Alfredo F. Fuentes–23,942–LPB	La Paz (Amor de Dios), Bolivia	3250	400–600
SH	*S. miniatum* (Herb.) Ravenna	Leaves	AF	Alfredo F. Fuentes–26,137–LPB	La Paz (Cota Cota), Bolivia	3400	500–700
SB	*S. miniatum* (Herb.) Ravenna	Bulbs	AF	Alfredo F. Fuentes–26,137–LPB	La Paz (Cota Cota), Bolivia	3400	500–700

**Table 2:** Alkaloid extraction yield from bulb and leaf samples of *P. boliviense* and *S. miniatum*, calculated as the percentage of alkaloid extract obtained relative to the crude extract.

Code	Dry Weight (DW, g)	Crude Extract Weight (g)	Alkaloid Extract Weight (g)	Yield (%)
PH	1.00	0.21	0.007	3.33
PB	1.00	0.34	0.004	1.18
SH	1.00	0.23	0.005	2.17
SB	1.08	0.55	0.008	1.46

Note: Yield (%) was determined as (alkaloid extract weight/crude extract weight) × 100.

**Table 3:** Alkaloid profile and individual alkaloid concentration values of *P. boliviese* and *S. miniatum* classified according to skeleton types proposed by [9]. Values are expressed in µg Gal/100 mg of dry weight (DW). **PH**, *P. boliviense* leaves; **PB**, *P. boliviense* bulbs; **SH**, *S. miniatum* leaves; **SB**, *S. miniatum* bulbs.

Alkaloids	RI	RT	[M^+^]	PH	PB	SH	SB
**Lycorine** **-type (total)**				**11.08**	**21.18**	**-**	**6.56**
Anhydrolycorine	2488.4	24.2117	251	-	3.34	-	-
Hippamine	2649.7	26.6600	301	-	3.76	-	-
Sternbergine	2682.3	27.1535	331	-	3.14	-	-
1-*O*-Acetyllycorine	2690.9	27.2843	329	-	3.13	-	-
Lycorine	2726.5	27.8235	287	5.82	4.59	-	-
Incartine	2721.2	27.7426	333	-	-	-	3.28
11,12-Dehydroanhydrolycorine	2594.9	25.8312	249	5.26	3.22	-	3.28
**Homolycorine-type (total)**				**-**	**6.93**	**92.09**	**15.28**
Homolycorine	2775.7	28.5685	109	-	3.14	92.09	5.45
Nerinine	2477.9	24.0459	109	-	3.79	-	9.83
**Galanthindole-type (total)**				**-**	**-**	**5.66**	**3.35**
Galanthindole	2485.9	24.1719	281	-	-	5.66	3.35
**Crinine / Haemanthamine-type (total)**				**10.51**	**9.50**	**25.06**	**13.49**
Vittatine/Crinine	2463.6	23.8204	271	-	-	5.69	5.22
8-*O*-Demethylmaritidine	2490.3	24.2407	273	5.27	3.24	3.48	3.56
Augustine	2556.1	25.2439	301	-	-	3.56	-
Crinamine	2620.0	26.2111	301	-	-	4.32	-
Haemanthamine	2614.8	26.1320	301	-	3.13	4.32	4.71
3-*O*-Acetylpowelline	2664.8	26.8889	343	-	-	3.69	-
Haemanthidine	2698.2	27.3948	317	5.24	3.13	-	-
**Narciclasine-type (total)**				**5.37**	**3.16**	**4.12**	**3.32**
Trisphaeridine	2276.0	20.8533	223	5.37	3.16	4.12	3.32
**Pretazettine-type (total)**					**3.16**	**28.15**	**14.42**
Tazettine	2627.2	26.3203	331	-	3.16	24.03	7.50
Epimacronine	2788.2	28.7571	329	-	-	4.12	3.37
*O*-Methyltazettine	2586.7	25.7065	345	-	-	-	3.55
**Montanine-type (total)**					**3.12**	**-**	**-**
Pancratinine C	2568.3	25.4283	287	-	3.12	-	-
**Galanthamine-type (total)**				**20.34**	**14.29**	**-**	**3.36**
Galanthamine	2384.9	22.5823	287	9.33	4.74	-	3.36
*N*-Demethylgalanthamine	2426.1	23.2307	273	5.70	3.28	-	-
Narwedine	2462.3	23.8003	285	5.31	3.15	-	-
3*-O-*Acetylgalanthamine	2516.8	24.6489	329	-	3.12	-	-
**Ismine-type (total)**					**6.25**	**4.42**	**5.24**
Demethylismine	2240.5	20.2740	243	-	3.13	-	-
Ismine	2256.5	20.5342	257	-	3.12	4.42	5.24
**Other type of alkaloids (total)s**				**10.95**	**13.08**	**-**	**3.34**
Tyramine	1418.9	8.2914	137	5.64	-	-	-
*N*-Methyltyramine	1456.5	8.6698	151	-	3.13	-	-
Hordenine	1464.6	8.7505	165	-	3.15	-	-
Demethylmesembrenol	2291.2	21.1017	275	5.31	3.68	-	-

**Table 4:** AChE and BuChE inhibitory activity of Bolivian species alkaloid extracts. Activity is expressed as the average of the values of IC_50_ (μg·mL^−1^) with standard deviations (SD). “NA” indicates that there is no inhibitory activity for the specific enzyme in the corresponding species. Galantamine (GAL) was included as a positive control. **PH**, *P. boliviense* leaves; **PB**, *P. boliviense* bulbs; **SH**, *S. miniatum* leaves; **SB**, *S. miniatum* bulbs.

	AChE (IC_50_)		BuChE (IC_50_)	
Sample	μg·mL^−1^ ± SD	Adjusted *p*-Value	μg·mL^−1^ ± SD	Adjusted *p*-Value
PH	24.86 ± 1.53	<0.001	NA	—
PB	6.07 ± 0.47	<0.001	30.93 ± 1.17	<0.01
SH	49.46 ± 2.07	<0.001	NA	—
SB	32.93 ± 3.92	<0.001	NA	—
GAL	0.35 ± 0.07		3.58 ± 0.14	

**Table 5:** In vitro anti-*T. cruzi* activity of alkaloid extracts from *P. boliviense* and *S. miniatum* against *T. cruzi* and cytotoxicity on Vero cells. **PH**, *P. boliviense* leaves; **PB**, *P. boliviense* bulbs; **SH**, *S. miniatum* leaves; **SB**, *S. miniatum* bulbs; NT, Not Tested. BNZ was used as a positive control. BNZ* values are expressed as µM.

	Vero Cytotoxicity	Anti-*T. cruzi* Assay		Anti-Amastigote Assay	
Sample	TC_50_ (μg·mL^−1^)	IC_50_ (μg·mL^−1^)	SI	IC_50_ (μg·mL^−1^)	SI
PH	NT	18.50 ± 28.71	—	NT	—
PB	23.95 ± 3.29	1.25 ± 0.51	19.11	9.67 ± 2.72	2.48
SH	16.53 ± 4.84	2.22 ± 1.03	7.45	NT	—
SB	18.14 ± 2.08	0.90 ± 0.15	20.12	1.42 ± 0.66	12.77
BNZ	>52.05	0.57 ± 0.04	>90	0.49 ± 0.01	>100
BNZ*	>200	2.2 ± 0.17	>90	1.88 ± 0.05	>100

## Data Availability

The original contributions presented in this study are included in the article. Further inquiries can be directed to the corresponding authors.

## Abbreviations

The following abbreviations are used in this manuscript:

AA	Amaryllidaceae alkaloids
AD	Alzheimer’s disease
AChE	Acetylcholinesterase
BuChE	Butyrylcholinesterase
AA	Alkaloids of Amaryllidaceae
CD	Chagas disease
FBS	Fetal bovine serum
DMSO	Dimethyl sulfoxide
MOI	Multiplicity of infection
CPRG	Chlorophenol red-β-D-galactopyranoside
